# Exploring Patient-Reported Gingival Health in Adults Using Standardised International FDI Oral Health Observatory Data

**DOI:** 10.1016/j.identj.2025.109320

**Published:** 2025-12-12

**Authors:** Tom Broomhead, Jennifer Kettle, Steve Mason, Sarah Baker

**Affiliations:** a Lincoln Institute for Rural and Coastal Health, University of Lincoln, Lincolnshire, UK; b School of Education, University of Sheffield, Sheffield, UK; c Haleon Research & Development (formerly GlaxoSmithKline Consumer Healthcare R&D), Weybridge, UK

**Keywords:** Global health, Periodontitis, Gingivitis, Social determinants of health, Wellbeing, Life satisfaction

## Abstract

**Background:**

Periodontitis is known to negatively affect oral health-related quality of life. Fewer studies have considered self-reported impacts of gingival health, despite evidence it can affect most people’s daily lives. Data limitations have also meant it was previously not possible to assess self-perceived gum health internationally. This study aimed to explore differences in the associations between gum health, socio-demographics, measures of health, wellbeing and oral health-related impacts in six countries using standardised international datasets among a nonprobabilistic sample of patients attending dental services.

**Methods:**

Linked patient-reported and dentist-reported data were collected from the World Dental Federation (FDI) Oral Health Observatory. Descriptive statistics and chi-square tests were used to analyse data from China (n = 2241), Colombia (n = 1029), India (n = 999), Italy (n = 711), Japan (n = 1271) and Lebanon (n = 798). Prevalence of patients reporting spitting or seeing blood when brushing and categorical periodontal status were the dependent variables, with age, sex, education, self-rated oral and general health, wellbeing, life satisfaction and oral health-related impacts included as independent variables.

**Results:**

Spitting or seeing blood when brushing was associated with education in five countries, while dentist-reported periodontal status worsened with age, lower education levels and among males. Worsening of both dependent variables was associated with poorer self-rated oral and general health in all countries, having a greater effect on oral health. Mixed results were seen for the association between spitting and seeing blood and wellbeing. In all countries worsening of the dependent variables was associated with life being less satisfying. Similar patterns were seen with oral health-related impacts in most cases. Country-specific patterns and variations were also detected.

**Conclusion:**

The exploratory findings can act as a basis for further research into country-specific patterns which are important for contextualising the findings, and for advocacy and understanding gingival health-related impacts and needs of patients in the countries investigated to date.

**Clinical relevance:**

This study found a number of associations between both patient and dentist-reported gum health and socio-demographic variables, measures of wellbeing, life satisfaction and oral health-related impacts. In particular, the importance of considering patient-reported outcomes and effects on daily life should be considered alongside clinical variables.

## Introduction

Periodontitis has been shown to negatively affect quality of life^1^^,^^2^ and has been associated with functional limitations and pain,^3^ as well as impacts associated with physical and psychological discomfort, psychological disability, social disability and physical disability.^4^^,^^5^ Yin and colleagues also categorised the impacts of periodontitis in terms of pressure (physical, psychosocial, financial), coping and adapting (avoidance, trying to learn more about their condition, taking responsibility) and reflection and evaluation (exploring the causes, personal control, calling for better dental care). Symptoms such as gingival recession,^6^^,^^7^ periodontal pockets and attachment loss^8^^,^^9^ contribute to decreases in oral health-related quality of life, which can affect the appearance, self-esteem and general health of patients with more severe symptoms.^10^

Fewer studies have considered the impacts of gingivitis, despite evidence that it can also affect quality of life and impact on daily life.^11^^,^^12^ Previous research has also demonstrated this to be the case across age ranges,^13^ including impacts on symptoms, function and emotional and social wellbeing in children^14^ and pain, difficulty brushing and wearing dentures in adults.^3^ ‘Gum health’ exists along a continuum^11^^,^^12^ and considering gum-related experiences from across this continuum is vital in understanding person-centred perspectives and developing new clinical intervention strategies. The importance of considering needs identified by patients and clinicians in developing communication and treatment plans has also been emphasised.^3^

To date, there have been very few multination studies of oral health outcomes, partly due to the cost and difficulty of organising oral health surveys and standardised data collection approaches internationally.^15^^,^^16^ Notable exceptions include studies on oral health-related behaviours^17^, ^18^, ^19^, ^20^ and dental attendance^21^ by welfare regimes using cross-national survey data, and associations between dmft, quality of life and a series of structural determinants in 11 countries.^22^ However, the use of existing secondary data sources (national samples or survey data) can have limitations.^23^ Standardised international primary research data can help to evaluate and plan oral health policies and services, while also allowing for comparisons of the impacts of policies, and benchmarking of oral health services for future advocacy purposes.^24^

In order to enable the collection of oral health data in a standardised, international manner, the World Dental Federation (FDI) and the International Consortium for Health Outcomes Measurement (ICHOM) developed the FDI-ICHOM Adult Oral Health Standard Set (AOHSS), a set of oral health measures which cover a comprehensive range of patient-centred oral health-related outcomes.^16^ Concurrently, FDI established the Oral Health Observatory (OHO), with the goal of collecting standardised, reliable and robust oral health-related datasets from primary dental care settings internationally. This data set comprised of patient-reported, clinical (dentist-reported) and dental practice level variables. The main aims of the project were to assist with advocacy at the national level, and via National Dental Associations (NDAs) help plan and optimise service provision and influence policy and investment, leading to improvements in oral health outcomes.^25^ An additional goal was to evaluate the concept of collecting standardised data from dental practices and patients for multination studies.

The OHO data is hugely beneficial in that, for the first time, it enables the evaluation of oral health from the perspective of both patients and dentists, combining self-rated outcomes with dentist-reported clinical data. This also allows an understanding of patterns of oral health and dental services at national level from both service users and providers while combining these with data about dental practices and service delivery. This in turn allows consideration of the effects of oral health, potential treatment needs and how the characteristics of services may affect patients’ oral health and related behaviours. The OHO also offers an inexpensive data collection method to fill gaps left when more costly national surveys are not possible. The OHO data is also available for a set of countries which are not typically included in the existing literature.

The aim of this study was to describe the prevalence and socio-demographics associated with ‘gum health’ across six countries (China, Colombia, India, Italy, Japan and Lebanon) among a nonprobabilistic sample of patients attending general dental services in primary care, and to explore the differences in self-rated and clinician-reported gingival health measures and their associations with other measures of health, wellbeing, life satisfaction and other oral health-related impacts.

## Methods

### Study design

The data were taken from a cross-sectional observational study of patients attending general dental services in primary care. A mobile app containing two questionnaires was used to generate data in dental practices, with one questionnaire completed by the patient and the other by the dentist about the patient’s clinical oral health status (which included a clinical examination). Both questionnaires were translated into the appropriate languages by professionals, before being verified by NDA staff who were fluent in English. Details on questionnaire development have been reported elsewhere.^26^

Stratified cluster sampling was used to select dentists, with registered dentists clustered by the administrative units (eg, province, state) they were located in. The proportion of the national population in each cluster determined the number of dentists to be recruited. Dentists were then randomly selected per cluster, with the aim of recruiting a minimum of 24 dentists per country (in line with commonly used sample sizes in feasibility studies not seeking to estimate effect size^27^).

A modified systematic sampling method was used for patient recruitment during the study period. One patient was surveyed each working day, based on the order in which they arrived at the practice; on the first day the first patient was surveyed, on the second day the second patient was surveyed, etc. If the selected patient declined, the following patient was invited to participate. This approach helped to minimise the risk of error or dentist dropouts. Fifty patients per dentist were surveyed and had to be able to give informed consent and reside in the study country. Parents gave proxy consent for children under the age of 12. Participating dentists were sent a guidance sheet, with patients receiving an information sheet.^26^

Patients completed the questionnaire using a tablet in clinic waiting rooms before their appointment, while dentists completed their questionnaire during an appointment or using patient records afterwards. Data were encrypted on the app and transferred to FDI’s secure servers. The datasets were linked by participant IDs.^26^

The OHO project was open to FDI member NDAs who had the capacity to implement the study protocol (countries without an FDI member NDA, or where the NDA was not willing or able to participate were ineligible). Countries meeting these criteria were able to express an interest in being part of the project. Twelve countries were involved in the initial phase,^26^ and the six included countries (who were the most advanced in their data collection before this paused due to Covid-19) were China, Colombia, India, Italy, Japan and Lebanon.

### Ethics

Ethical reviews were undertaken and approved both in Japan and Lebanon (Niigata University Ethics Review Board, application 2017-0285; Lebanese Dental Association Ethics Review Board, application 54ETH/19). In China, Colombia, India and Italy, the project was reviewed in line with national regulations, and as such it was not necessary to submit to separate ethical review bodies. Participating patients received the study information sheet from their dentist, and consent was obtained through the mobile app prior to completion of the questionnaire.^28^

### ‘Gum health’

‘Gum health’ was measured using one patient-reported variable and one dentist-reported variable. Patients were asked ‘Do you spit blood or see blood when you brush your teeth?’ Responses were ‘yes’, ‘no’ and ‘rather not answer’. The subjective gum health measure was added to the OHO questionnaire by members of the research team from Haleon. Periodontal status was collected from the dentist questionnaire (involving a clinical examination) as a categorical variable, with the following response options: ‘healthy’, ‘gingivitis’, ‘pockets’ (both shallow and deep) and ‘mobile teeth’. This variable was recategorised into ‘healthy’, ‘gingivitis’ and ‘periodontitis’ (combining pockets and mobile teeth). This categorisation is in line with guidelines established by the European Federation of Periodontology,^29^ which classified periodontal diagnosis into periodontal health, gingivitis and periodontitis (including measures of pocket depth and loss of support, resulting in mobile teeth). Only patients who had been clinically assessed as part of the dentist questionnaire were able to be classified as having periodontitis. The full procedure for collecting clinical data as part of the OHO project has been published elsewhere.^28^ The clinical variable was based on the WHO Oral Health Survey Basic Methods document (Section 1.5.5.2).^26^^,^^28^^,^^30^ Dentists were instructed to follow this guidance, although no further formal training and calibration were conducted.

### Variables: Patient-reported

#### Socio-demographic variables

Age, sex and education were included as socio-demographic variables. Age was originally collected as a continuous variable and then recategorised into 18 to 34, 35 to 64 and 65+ years. Under 18s were excluded as part of this analysis. These age categories were used as they matched categorisations in a national decennial representative survey^31^ and allowed for distinction between younger adults (18-34) and older adults (65+). Sex was collected as female or male. Education was measured using the International Standard Classification of Education,^32^ which included 10 response options, and was recategorised into ‘higher education’, ‘secondary/further education’ and ‘no education/primary education’.

#### Self-rated oral health and general health

Self-rated oral health was measured using the question, ‘How would you rate your oral health?’ Responses for this variable were recorded on a 5-point Likert scale (very good to very poor). This variable was recategorised for the analysis into the following three categories: ‘very good/good’, ‘fair’, and ‘poor/very poor’. Self-rated general health was measured using the question, ‘How would you rate your general health?’ Responses were recorded on the same 5-point Likert scale and recategorised into the same three categories for analysis. These variables were chosen from existing validated scales and were voted on for inclusion by a panel of experts.^16^

#### Relationship between oral health and general wellbeing/satisfaction with life

Participants were asked how they perceived the relationship between their oral health and their general wellbeing and satisfaction with life. The relationship between oral health and general wellbeing was measured using the statement, ‘My oral health has a good impact on my general wellbeing’. Response options were ‘strongly agree’, ‘agree’, ‘disagree’ or ‘strongly disagree’ and these were recategorised into ‘strongly agree/agree’ and ‘disagree/strongly disagree’ for the analysis. The relationship between oral health and satisfaction with life was measured using the question, ‘Have you found that life in general was less satisfying due to problems with your mouth, teeth or dentures during the past 12 months?’ Response options were ‘yes’ or ‘no’. These measures of wellbeing and life satisfaction were part of a previous successful feasibility study, where the reliability of the OHO questionnaires and acceptability of the approach and methods used were established.^26^

#### Oral health-related impacts

In addition to spitting or seeing blood when brushing, eight items related to impacts associated with the mouth, teeth or dentures in the past 12 months were included: pain; discomfort; difficulty eating, chewing or biting food; difficulty speaking or pronouncing words; feeling embarrassed to smile or laugh; problems sleeping; limiting participation in social activities/difficulty enjoying contact with others; and difficulty carrying out work or major role. Response options were ‘yes’ or ‘no’, and participants were then asked to rate how much the item had affected them on a scale of 1 to 5, with 1 being ‘not at all’ and 5 being ‘very much’. These variables were chosen from existing validated scales and were voted on for inclusion by a panel of experts.^16^

### Data analysis

The same analysis was carried out separately on the data from each of the six countries, in order to explore the patterns in each country individually as has been done in previous studies.^28^^,^^33^ Descriptive statistics were gathered on the patient-reported measure of spitting or seeing blood when brushing and the dentist-reported measure of periodontal status. Chi-square tests of independence were used to establish whether participant experiences of spitting or seeing blood when brushing and periodontal status were associated with the socio-demographic, self-rated oral and general health, wellbeing and life satisfaction and oral health-related impact variables. A 95% significance level was used throughout. Analyses were conducted using IBM SPSS Statistics (Version 28).^34^

## Results

There were 7049 participants across the six countries as follows: China (n = 2241), Colombia (n = 1029), India (n = 999), Italy (n = 711), Japan (n = 1271) and Lebanon (n = 798).

### Prevalence of spitting or seeing blood, and periodontal status

Thirty-two percent of participants experienced spitting or seeing blood when they brushed, ranging from 20.4% in Colombia to 40.9% in China (Table 1). The proportion of participants with healthy periodontal status was 40.4%, compared to 32.7% with gingivitis and 26.9% with periodontitis (24.3% of participants had shallow or deep pockets and 2.6% had mobile teeth). Patients could only be classified as having periodontitis through the clinical examination in the dentist’s questionnaire. The proportion with healthy periodontal status ranged from 21.1% in Japan to 53.1% in India (Table 2). Spitting or seeing blood when brushing was significantly associated with periodontal status in all six countries (Table 3). Overall, 14.7% of participants with healthy teeth, 38.3% of participants with gingivitis and 50.3% of patients with periodontitis experienced spitting or seeing blood when brushing.

**Table 1:** Spitting or seeing blood, prevalence.

	China		Colombia		India		Italy		Japan		Lebanon	
Spitting or seeing blood when brushing	N	%	N	%	N	%	N	%	N	%	N	%
Yes	916	40.9	210	20.4	253	25.3	173	24.3	465	36.6	240	30.1
No	1326	59.1	819	79.6	746	74.7	539	75.7	806	63.4	558	69.9
**Total**	**2242**	**100.0**	**1029**	**100.0**	**999**	**100.0**	**711**	**100.0**	**1271**	**100.0**	**798**	**100.0**

**Table 2:** Periodontal status, prevalence.

	China		Colombia		India		Italy		Japan		Lebanon	
Periodontal status	N	%	N	%	N	%	N	%	N	%	N	%
Healthy	832	37.7	537	52.3	529	53.1	306	43.7	262	21.1	344	43.5
Gingivitis	801	36.3	381	37.1	244	24.5	206	29.4	322	25.9	324	41.0
Shallow pocket	375	17.0	86	8.4	113	11.3	137	19.5	431	34.6	87	11.0
Deep pocket	140	6.4	9	0.9	61	6.1	41	5.8	186	15.0	25	3.2
Mobile teeth	56	2.5	13	1.3	49	4.9	11	1.6	43	3.5	10	1.3
**Total**	**2204**	**98.3**	**1026**	**99.7**	**996**	**99.7**	**701**	**98.6**	**1244**	**97.9**	**790**	**99.0**
Missing	38	1.7	3	0.3	3	0.3	10	1.4	27	2.1	8	1.0
**Total**	**2242**	**100.0**	**1029**	**100.0**	**999**	**100.0**	**711**	**100.0**	**1271**	**100.0**	**798**	**100.0**

**Table 3:** Spitting or seeing blood and periodontal status, cross tabulation.

		Experienced spitting/seeing blood when brushing					
		China	Colombia	India	Italy	Japan	Lebanon
		Y % (n)	Y % (n)	Y % (n)	Y % (n)	Y % (n)	Y % (n)
**Periodontal status**	Healthy	22.2 (185)	5.2 (28)	12.5 (66)	15.0 (46)	22.9 (60)	7.8 (27)
	Gingivitis	46.1 (369)	31.5 (120)	30.7 (75)	28.2 (58)	35.4 (114)	42.0 (136)
	Periodontitis	60.1 (343)	55.6 (60)	50.2 (112)	36.5 (69)	42.7 (282)	63.1 (77)
	**Chi-square (df, *P* value)**	**215.9 (2, *P* < .001) Missing: 38**	**188.2 (2, *P* < .001) Missing: 3**	**122.8 (2, *P* < .001) Missing: 3**	**30.9 (2, *P* < .001) Missing: 10**	**32 (2, *P* < .001) Missing: 27**	**165 (2, *P* < .001) Missing: 8**

Note: Significant ***P*** values are shown in bold.

### Socio-demographic differences

Spitting or seeing blood when brushing was significantly associated with education in five countries (China, Colombia, India, Japan and Lebanon), with higher proportions of this symptom among those with no education or primary education in Colombia, Japan and Lebanon, higher education in India and secondary or further education in China. Significant associations with age were found in China (highest among the 35-64 age group), Japan (18-34 and 35-64 age group) and Lebanon (35-64 and 65+ age group). Significant associations with sex were also found among male participants in China and Japan, and among female participants in Colombia (Table 4).

**Table 4:** Periodontal status, spitting or seeing blood and age, sex and education, cross tabulation.

		China				Colombia				India				Italy				Japan				Lebanon			
		**Healthy% (n)**	**Gingivitis% (n)**	**Periodontal disease % (n)**	**Spitting, seeing blood % (n)**	**Healthy% (n)**	**Gingivitis% (n)**	**Periodontal disease % (n)**	**Spitting, seeing blood % (n)**	**Healthy% (n)**	**Gingivitis% (n)**	**Periodontal disease % (n)**	**Spitting, seeing blood % (n)**	**Healthy% (n)**	**Gingivitis% (n)**	**Periodontal disease % (n)**	**Spitting, seeing blood % (n)**	**Healthy% (n)**	**Gingivitis% (n)**	**Periodontal disease % (n)**	**Spitting, seeing blood % (n)**	**Healthy% (n)**	**Gingivitis% (n)**	**Periodontal disease % (n)**	**Spitting, seeing blood % (n)**
**Age**	18-34	48.5 (569)	37.8 (444)	13.7 (161)	35.5 (424)	61.2 (322)	35.4 (186)	3.4 (18)	19.3 (102)	58.1 (258)	29.7 (132)	12.2 (54)	26.7 (119)	56.6 (125)	33.0 (73)	10.4 (23)	27.4 (61)	43.8 (95)	38.7 (84)	17.5 (38)	39.5 (87)	52.8 (197)	42.6 (159)	4.6 (17)	25.7 (97)
	35-64	26.3 (241)	35.7 (327)	37.9 (347)	48.8 (455)	45.9 (195)	40.9 (174)	13.2 (56)	21.4 (91)	50.2 (248)	21.1 (104)	28.7 (142)	24.8 (123)	40.6 (153)	30.2 (114)	29.2 (110)	24.2 (93)	19.5 (124)	26.8 (170)	53.7 (341)	39.2 (255)	35.3 (127)	39.4 (142)	25.3 (91)	34.0 (123)
	65+	19.1 (22)	26.1 (30)	54.8 (63)	31.9 (37)	26.7 (20)	28.0 (21)	45.3 (34)	22.7 (17)	39.7 (23)	13.8 (8)	46.6 (27)	19.0 (11)	27.2 (28)	18.4 (19)	54.4 (56)	18.3 (19)	11.0 (43)	17.3 (68)	71.7 (281)	30.7 (123)	35.1 (20)	40.4 (23)	24.6 (14)	34.5 (20)

	**Chi-square (df, *P* value)**	**237.4 (4,***P***< .001) Missing: 38**			**41.9 (2,***P***< .001) Missing: 0**	**138.9 (4,***P***< .001) Missing: 3**			.9 (2, *P* = .650) Missing: 0	**59.7 (4,***P***< .001) Missing: 3**			1.7 (2, *P* = .420) Missing: 0	**72.8 (4,***P***< .001) Missing: 10**			3.2 (2, *P* = .203) Missing: 0	**175.2 (4,***P***< .001) Missing: 27**			**8.8 (2,***P***= .012)** **Missing: 0**	**68.6 (4,***P***< .001) Missing: 8**			**6.6 (2,***P***= .036) Missing: 0**

**Sex**	Female	42.7 (541)	35.3 (448)	22.0 (279)	37.2 (481)	46.9 (229)	40.6 (198)	12.5 (61)	23.9 (117)	44.8 (171)	30.6 (117)	24.6 (94)	24.0 (92)	50.0 (199)	27.6 (110)	22.4 (89)	23.3 (95)	23.9 (170)	28.6 (203)	47.5 (337)	33.6 (243)	42.9 (171)	41.9 (167)	15.3 (61)	28.9 (116)
	Male	31.1 (288)	37.6 (348)	31.2 (289)	45.9 (431)	57.4 (308)	33.9 (182)	8.8 (47)	17.1 (92)	58.3 (358)	20.7 (127)	21.0 (129)	26.1 (161)	35.1 (106)	31.8 (96)	33.1 (100)	25.7 (78)	17.0 (88)	22.2 (115)	60.8 (315)	40.6 (216)	44.1 (171)	40.2 (156)	15.7 (61)	31.6 (124)

	**Chi-square (***P***value)**	**37.2 (2,***P***< .001)** **Missing: 49**			**16.9 (1,***P***< .001) Missing: 11**	**11.8 (2,***P***= .003)** **Missing: 4**			**7.3 (1,***P***= .007)** **Missing: 1**	**19 (2,***P***< .001)** **Missing: 3**			.6 (1, *P* = .455) Missing = 0	**17.1 (2,***P***< .001)** **Missing: 11**			.5 (1, *P* = .461) Missing: 1	**21.7 (2,***P***< .001)** **Missing: 43**			**6.5 (1,***P***= .011)** **Missing: 16**	.2 (2, *P* = .895) Missing: 11			.7 (1, *P* = .408) Missing: 3

**Education**	No education/primary education	23.3 (28)	24.2 (29)	52.5 (63)	37.4 (46)	25.7 (28)	37.6 (41)	36.7 (40)	32.1 (35)	65.1 (185)	13.7 (39)	21.1 (60)	27.7 (79)	30.0 (15)	24.0 (12)	46.0 (23)	28.0 (14)	20.0 (4)	30.0 (6)	50.0 (10)	38.1 (8)	25.0 (19)	50.0 (38)	25.0 (19)	36.4 (28)
	Secondary/further education	32.5 (269)	35.1 (290)	32.4 (268)	45.9 (384)	55.7 (376)	36.6 (247)	7.7 (52)	18.8 (126)	57.3 (211)	21.2 (78)	21.5 (79)	20.3 (75)	40.7 (206)	30.8 (156)	28.5 (144)	24.9 (128)	18.6 (137)	24.5 (180)	56.9 (418)	37.0 (279)	37.5 (101)	43.9 (118)	18.6 (50)	34.7 (94)
	Higher education	42.4 (505)	38.2 (455)	19.5 (232)	37.9 (461)	54.6 (131)	38.8 (93)	6.7 (16)	20.0 (48)	38.3 (125)	38.0 (124)	23.6 (77)	29.4 (96)	58.6 (82)	26.4 (37)	15.0 (21)	22.0 (31)	26.0 (119)	28.0 (128)	46.0 (210)	35.4 (164)	50.7 (224)	37.3 (165)	12.0 (53)	26.2 (117)
	**Chi-square (***P***value)**	**90 (4,***P***< .001) Missing: 103**			**13.7 (2,***P***= .001)** **Missing: 66**	**96 (4,***P***< .001) Missing: 5**			**10.6 (2,***P***= .005)** **Missing: 2**	**62 (4,***P***< .001] Missing: 21**			**8.7 (2,***P***= .013)** **Missing: 18**	**26.1 (4,***P***< .001) Missing: 15**			.9 (2, *P* = .654) Missing: 5	**15.1 (4,***P*= .004)* Missing: 59			**10.6 (2,***P***= .005)** **Missing: 33**	**26.4 (4,***P***< .001) Missing: 11**			**7.4 (2,***P***= .024)** **Missing: 3**

Note: Significant *P* values are shown in bold

Periodontal status was significantly associated with age in all six countries, with increased age being associated with periodontitis (with the exception Lebanon where similar proportions were found for the 35-64 and 65+ age groups). The proportion of participants with gingivitis also declined with age in all countries. Higher education was associated with better periodontal health, with higher proportions of these participants having healthy teeth compared to the proportion with gingivitis and periodontitis. The exception was Japan, where higher proportions of participants with higher education had gingivitis and periodontitis compared to healthy teeth. Higher education was also significantly associated with lower proportions of gingivitis and periodontitis compared to participants with no more than primary education. The exception to this was India, where the proportion of patients with healthy teeth declined as education level increased, while the proportions with gingivitis and periodontitis increased with higher levels of education.

In relation to sex, the picture was mixed. In China and Italy, higher proportions of females than males had healthy teeth and lower proportions had gingivitis and periodontitis. In Japan, a higher proportion of females than males had healthy teeth and gingivitis, but a lower proportion had periodontitis. In Colombia and India, higher proportions of females than males had gingivitis and periodontitis and lower proportions had healthy teeth.

### Association with self-rated oral health and general health

Spitting or seeing blood was significantly associated with poorer self-rated oral health and poorer self-rated general health in all six countries (higher proportions of participants who experienced this rated their oral and general health as poor or very poor). Patterns varied by country with regard to those responding ‘fair’. There were also stronger relationships between spitting or seeing blood and oral health compared to general health, with higher proportions of those experiencing these symptoms rating their oral health as poor/very poor and lower proportions rating this as good/very good compared to general health (Table 5).

**Table 5:** Periodontal status, spitting or seeing blood, self-rated oral health and general health, cross tabulation.

		Self-rated oral health																	
		China			Colombia			India			Italy			Japan			Lebanon		
		Very good/good % (n)	Fair % (n)	Poor/very poor % (n)	Very good/good % (n)	Fair % (n)	Poor/very poor % (n)	Very good/good % (n)	Fair % (n)	Poor/very poor % (n)	Very good/good % (n)	Fair % (n)	Poor/very poor % (n)	Very good/good % (n)	Fair % (n)	Poor/very poor % (n)	Very good/good % (n)	Fair % (n)	Poor/very poor % (n)
**Periodontal status**	Healthy	56.0 (464)	36.2 (300)	7.7 (64)	54.2 (289)	43.3 (231)	2.4 (13)	62.2 (327)	23.0 (121)	14.8 (78)	62.2 (189)	31.9 (97)	5.9 (18)	33.1 (86)	53.8 (140)	13.1 (34)	74.7 (257)	20.9 (72)	4.4 (15)
	Gingivitis	46.7 (373)	43.7 (349)	9.6 (77)	54.1 (203)	28.3 (106)	17.6 (66)	39.8 (97)	48.4 (118)	11.9 (29)	40.9 (83)	50.7 (103)	8.4 (17)	14.4 (46)	58.8 (188)	26.9 (86)	50.6 (164)	34.6 (112)	14.8 (48)
	Periodontal disease	20.5 (117)	52.1 (297)	27.4 (156)	28.0 (30)	10.3 (11)	61.7 (66)	22.9 (51)	44.8 (100)	32.3 (72)	27.1 (51)	54.8 (103)	18.1 (34)	9.1 (60)	47.7 (313)	43.1 (283)	34.4 (42)	34.4 (42)	31.1 (38)
	**Chi-square (df,***P***value)**	**231.3 (4,***P***< .001) Missing: 45**			**270.9 (4,***P***< .001) Missing: 14**			**129.3 (4,***P***< .001) Missing: 6**			**67.9 (4,***P***< .001) Missing: 16**			**130.3 (4,***P***< .001) Missing: 35**			**95.8 (4,***P***< .001) Missing: 8**		
	Spitting, seeing blood % (n)	30.8 (281)	47.9 (436)	21.3 (194)	41.3 (85)	22.3 (46)	36.4 (75)	26.1 (66)	45.8 (116)	28.1 (71)	30.2 (51)	55.6 (94)	14.2 (24)	9.1 (42)	42.4 (196)	48.5 (224)	40.8 (98)	36.3 (87)	22.9 (55)
	**Chi-square (df,***P***value)**	**128.6 (2,***P***< .001) Missing: 7**			**105 (2,***P***< .001) Missing: 11**			**66.5 (2,***P***< .001) Missing: 3**			**25.2 (2,***P***< .001) Missing: 6**			**91.3 (2,***P***< .001) Missing: 9**			**53 (2,***P***< .001) Missing: 0**		


		Self-rated general health																	
		China			Colombia			India			Italy			Japan			Lebanon		
		Very good/good % (n)	Fair % (n)	Poor/very poor % (n)	Very good/good % (n)	Fair % (n)	Poor/very poor % (n)	Very good/good % (n)	Fair % (n)	Poor/very poor % (n)	Very good/good % (n)	Fair % (n)	Poor/very poor % (n)	Very good/good % (n)	Fair % (n)	Poor/very poor % (n)	Very good/good % (n)	Fair % (n)	Poor/very poor % (n)
**Periodontal status**	Healthy	66.0 (548)	31.6 (262)	2.4 (20)	53.7 (286)	43.5 (232)	2.8 (15)	63.8 (337)	17.6 (93)	18.6 (98)	68.0 (208)	28.8 (88)	3.3 (10)	32.7 (85)	56.9 (148)	10.4 (27)	87.2 (299)	11.1 (38)	1.7 (6)
	Gingivitis	60.0 (479)	36.5 (291)	3.5 (28)	57.1 (216)	29.1 (110)	13.8 (52)	44.3 (108)	43.9 (107)	11.9 (29)	63.4 (130)	33.7 (69)	2.9 (6)	24.7 (79)	65.0 (208)	10.3 (33)	71.9 (233)	22.2 (72)	5.9 (19)
	Periodontal disease	43.3 (247)	46.7 (266)	10.0 (57)	39.6 (42)	15.1 (16)	45.3 (48)	30.0 (67)	55.6 (124)	14.3 (32)	43.4 (82)	50.8 (96)	5.8 (11)	16.3 (107)	69.5 (456)	14.2 (93)	50.8 (62)	40.2 (49)	9.0 (11)
	**Chi-square (df,***P***value)**	**96.8 (4,***P***< .001) Missing: 44**			**175.6 (4,***P***< .001) Missing: 12**			**125.3 (4,***P***< .001) Missing: 4)**			**31.2 (4,***P***< .001) Missing: 11**			**32.5 (4,***P***< .001) Missing: 35**			**68 (4,***P***< .001) Missing: 9**		
	Spitting, seeing blood % (n)	49.9 (455)	42.9 (391)	7.2 (66)	52.7 (109)	20.3 (42)	27.1 (56)	36.4 (92)	44.3 (112)	19.4 (49)	48.6 (84)	45.7 (79)	5.8 (10)	16.0 (74)	66.7 (309)	17.3 (80)	65.0 (156)	30.4 (73)	4.6 (11)
	**Chi-square (df,***P***value)**	**51.1 (2,***P***< .001) Missing: 6**			**73.6 (2,***P***< .001) Missing: 9**			**31.9 (2,***P***< .001) Missing: 1**			**13.9 (2,***P***< .001) Missing: 1**			**25.8 (2,***P***< .001) Missing: 8**			**21.3 (2,***P***< .001**) **Missing: 1**		

Note: Significant *P* values are shown in bold.

Periodontal status was also significantly associated with poorer self-rated oral health in all countries. The majority of participants with healthy teeth in China, Colombia, India, Italy and Lebanon rated their oral health as good or very good (in Japan the majority rated their oral health as fair). Compared to those with healthy teeth, higher proportions of participants with gingivitis rated their oral health as fair (except in Colombia), and in all countries oral health was rated as poor or very poor most commonly among those with periodontitis.

Similar patterns were found for general health, with the exception of India, where a higher proportion of those with healthy teeth rated their general health as poor or very poor compared to those with gingivitis and periodontitis. Stronger relationships were again seen between periodontal status and self-rated oral health compared to self-rated general health. In all countries, higher proportions of participants with gingivitis and periodontitis rated their oral health as poor or very poor compared to those rating their general health in the same way (except in India where the proportion for gingivitis was identical), with lower proportions rating their oral health as good or very good compared to general health.

### Association with good impact on wellbeing and less satisfaction with life

Spitting or seeing blood was significantly associated with general wellbeing in five countries (excluding Italy). In China, India and Lebanon, higher proportions of those experiencing these symptoms agreed that oral health had a good impact on their wellbeing, compared to Colombia and Japan, where the same group disagreed. Spitting or seeing blood was also significantly associated with less satisfaction in life in all countries, with higher proportions who had experienced these symptoms agreeing with this statement (Table 6).

**Table 6:** Periodontal status, spitting or seeing blood and impact on wellbeing/satisfaction with life, cross tabulation

		My oral health has a good impact on my general wellbeing.											
		China		Colombia		India		Italy		Japan		Lebanon	
		Strongly agree/agree % (n)	Disagree/strongly disagree % (n)	Strongly agree/agree % (n)	Disagree/strongly disagree % (n)	Strongly agree/agree % (n)	Disagree/strongly disagree % (n)	Strongly agree/agree % (n)	Disagree/strongly disagree % (n)	Strongly agree/agree % (n)	Disagree/strongly disagree % (n)	Strongly agree/agree % (n)	Disagree/strongly disagree % (n)
Periodontal status	Healthy	96.2	3.8	96.2	3.8	91.7	8.3	97.7	2.3	92.5	7.5	94.8	5.2
		(784)	(31)	(513)	(20)	(483)	(44)	(297)	(7)	(223)	(18)	(325)	(18)
	Gingivitis	94.9	5.1	86.3	13.7	96.6	3.4	97.6	2.4	85.8	14.2	94.0	6.0
		(746)	(40)	(316)	(50)	(228)	(8)	(200)	(5)	(247)	(41)	(298)	(19)
	Periodontal disease	96.0	4.0	84.9	15.1	94.0	6.0	97.8	2.2	88.6	11.4	94.2	5.8
		(529)	(22)	(90)	(16)	(202)	(13)	(182)	(4)	(531)	(68)	(113)	(7)
	Chi-square (df, p value)	1.8 (2, p = .408)		33.7 (2, p <.001)		6.6 (2, p = .036)		.04 (2, p = .982)*		6 (2, p = .049)		.2 (2, p = .913)	
		Missing = 90		Missing: 24		Missing: 21		Missing: 16		Missing: 143		Missing: 18	
	Spitting, seeing blood % (n)	96.7	3.3	85.9	14.1	97.1	2.9	96.5	3.6	84.4	15.6	97.1	2.9
		(863)	(29)	(164)	(27)	(237)	(7)	(166)	(6)	(352)	(65)	(743)	(45)
	Chi-square (df, p value)	4.3 (1, p = .038)		22.8 (1, p <.001)		7.4 (1, p = .006)		1.5 (1, p = .217)*		12 (1, p <.001)		4.9 (1, p = .028)	


		Life in general felt less satisfying due to problems with mouth or teeth in past 12 months.											
		China		Colombia		India		Italy		Japan		Lebanon	
		Y	N	Y	N	Y	N	Y	N	Y	N	Y	N
		% (n)	% (n)	% (n)	% (n)	% (n)	% (n)	% (n)	% (n)	% (n)	% (n)	% (n)	% (n)
Periodontal status	Healthy	19.4	80.6	8.8	91.2	22.2	77.8	25.8	74.2	10.9	89.1	23.8	76.2
		(156)	(647)	(46)	(475)	(117)	(410)	(78)	(224)	(28)	(228)	(80)	(256)
	Gingivitis	22.0	78.0	20.7	79.3	28.5	71.5	32.7	67.3	12.6	87.4	36.5	63.5
		(172)	(611)	(76)	(291)	(67)	(168)	(67)	(138)	(40)	(277)	(116)	(202)
	Periodontal disease	36.2	63.8	47.2	52.8	44.4	55.6	34.9	65.1	19.5	80.5	56.8	43.2
		(197)	(347)	(51)	(57)	(95)	(119)	(66)	(123)	(127)	(524)	(67)	(51)
	Chi-square (df, p value)	54 (2, p <.001)		96.4 (2, p <.001)		36.7 (2, p <.001)		5.3 (2, p = .070)		13.6 (2, p = .001)		43.7 (2, p <.001)	
		Missing = 112		Missing: 33		Missing: 23		Missing: 15		Missing: 47		Missing: 26	
	Spitting, seeing blood % (n)	32.0	68.0	35.9	64.1	53.7	46.3	37.4	62.6	22.3	77.7	51.9	48.1
		(281)	(596)	(74)	(132)	(132)	(114)	(64)	(107)	(103)	(358)	(267)	(513)
	Chi-square (df, p value)	42.8 (1, p <.001)		61.8 (1, p <.001)		101 (1, p <.001)		5.2 (1, p = .023)		232 (1, p <.001)		46.2 (1, p <.001)	

Significant p values are shown in bold

⁎ 2 cells have expected count less than 5. The minimum expected count is 4.28.

Periodontal status was also significantly associated with general wellbeing in Colombia, India and Japan. In Colombia and Japan, higher proportions with healthy teeth agreed that oral health had a good impact on their wellbeing, while in India higher proportions agreed among those with gingivitis and periodontitis. Periodontal status was associated with less satisfaction with life in five countries (excluding Italy), where higher proportions of participants with gingivitis and periodontitis agreed with this statement (compared to those with healthy teeth).

### Association with oral health-related impacts

Spitting or seeing blood was significantly associated with oral health-related impacts in the majority of cases. Higher proportions of participants experiencing these symptoms also experienced the full range of oral health-related impacts in China, Colombia, India and Lebanon. The only exceptions were limited participation in social activities in Italy, and difficulty carrying out one’s major work or role in Japan (Table 7).

**Table 7:** Periodontal status, spitting or seeing blood and oral health-related impacts, cross tabulation.

		**Experienced spitting/seeing blood when brushing**																	
		**China**			**Colombia**			**India**			**Italy**			**Japan**			**Lebanon**		
		**Y % (n)**	**N % (n)**	**Chi-square (df,***P***value)**	**Y % (n)**	**N % (n)**	**Chi-square (df,***P***value)**	**Y % (n)**	**N % (n)**	**Chi-square (df,***P***value)**	**Y % (n)**	**N % (n)**	**Chi-square (df,***P***value)**	**Y % (n)**	**N % (n)**	**Chi-square (df,***P***value)**	**Y % (n)**	**N % (n)**	**Chi-square (df,***P***value)**
		**Discomfort**			**Discomfort**			**Discomfort**			**Discomfort**			**Discomfort**			**Discomfort**		
**Experienced spitting/seeing blood when brushing**	Healthy	32.3 (268)	67.8 (564)	**114.8 (2,***P***< .001) Missing: 38**	17.3 (93)	82.7 (444)	**95.2 (2,***P***< .001) Missing: 3**	32.7 (173)	67.3 (356)	**51.8 (2,***P***< .001) Missing: 3**	41.5 (127)	58.5 (179)	**32.2 (2,***P***< .001) Missing: 10**	28.6 (75)	71.4 (187)	**27 (2,***P***< .001) Missing: 27**	27.9 (96)	72.1 (248)	**30.4 (2,***P***< .001) Missing: 8**
	Gingivitis	44.6 (357)	55.4 (444)		30.2 (115)	69.8 (266)		41.8 (102)	58.2 (142)		51.5 (106)	48.5 (100)		33.9 (109)	66.1 (213)		46.3 (150)	53.7 (174)	
	Periodontal disease	61.1 (349)	38.9 (222)		62.0 (67)	38.0 (41)		61.0 (136)	39.0 (87)		67.7 (128)	32.3 (61)		54.5 (360)	45.5 (300)		49.2 (60)	50.8 (62)	
	Spitting, seeing blood	60.3 (552)	39.7 (364)	**160.1 (1,***P***< .001**) **Missing: 0**	50.5 (106)	49.5 (104)	**76 (1,***P***< .001) Missing: 0**	68.0 (172)	32.0 (81)	**100.8 (1,***P***< .001) Missing: 0**	65.3 (113)	34.7 (60)	**18.9 (1,***P***< .001) Missing: 0**	49.7 (231)	50.3 (234)	**36.1 (1,***P***< .001) Missing: 0**	52.5 (126)	47.5 (114)	**26.4 (1,***P***< .001) Missing: 0**
		**Pain**			**Pain**			**Pain**			**Pain**			**Pain**			**Pain**		
	Healthy	23.9 (199)	76.1 (633)	**118.4(2,***P***< .001) Missing: 38**	11.4 (61)	88.6 (476)	**127.3 (2,***P***< .001) Missing: 3**	19.1 (101)	80.9 (428)	**123.2 (2,***P***< .001) Missing: 3**	29.1 (89)	70.9 (217)	**16.6 (2,***P***< .001) Missing: 10**	23.7 (62)	76.3 (200)	**21.3 (2,***P***< .001) Missing: 27**	36.0 (124)	64.0 (220)	**35.5 (2,***P***< .001) Missing: 8**
	Gingivitis	35.2 (282)	64.8 (519)		26.8 (102)	73.2 (279)		42.2 (103)	57.8 (141)		37.9 (78)	62.1 (128)		33.2 (107)	66.8 (215)		50.9 (165)	49.1 (159)	
	Periodontal disease	52.2 (298)	47.8 (273)		59.3 (64)	40.7 (44)		59.2 (132)	40.8 (91)		47.1 (89)	52.9 (100)		39.5 (261)	60.5 (399)		65.6 (80)	34.4 (42)	
	Spitting, seeing blood	48.9 (448)	51.1 (468)	**122.4 (1,***P***< .001) Missing: 0**	44.39 (93)	55.7 (117)	**75.8 (1,***P***< .001) Missing: 0**	65.2 (165)	34.8 (88)	**150.2 (1,***P***< .001) Missing: 0**	52.6 (91)	47.4 (82)	**26.3 (1,***P***< .001) Missing: 0**	48.8 (227)	51.2 (238)	**64.5 (1,***P***< .001) Missing: 0**	65.4 (157)	34.6 (83)	**48.7 (1,***P***< .001) Missing: 0**
		**Difficulty eating food**			**Difficulty eating food**			**Difficulty eating food**			**Difficulty eating food**			**Difficulty eating food**			**Difficulty eating food**		
	Healthy	16.5 (137)	83.5 (695)	**147.7 (2,***P***< .001) Missing: 38**	9.7 (52)	90.3 (485)	**126 (2,***P***< .001) Missing: 3**	25.0 (132)	75.0 (397)	**80.8 (2,***P***< .001) Missing: 3**	22.5 (69)	77.5 (237)	**22.3 (2,***P***< .001) Missing: 10**	17.2 (45)	82.8 (217)	**37.7 (2,***P***< .001) Missing: 27**	24.1 (83)	75.9 (261)	**70.5 (2,***P***< .001) Missing: 8**
	Gingivitis	23.0 (184)	77.0 (617)		21.8 (83)	78.2 (298)		38.5 (94)	61.5 (150)		38.3 (79)	61.7 (127)		21.4 (69)	78.6 (253)		35.2 (114)	64.8 (210)	
	Periodontal disease	44.8 (256)	55.2 (315)		55.6 (60)	44.4 (48)		59.2 (132)	40.8 (91)		40.2 (76)	59.8 (113)		34.8 (230)	65.2 (430)		66.4 (81)	33.6 (41)	
	Spitting, seeing blood	36.6 (335)	63.4 (581)	**84.8 (1,***P***< .001) Missing: 0**	41.4 (87)	58.6 (123)	**86.8 (1,***P***< .001) Missing: 0**	64.4 (163)	35.6 (90)	**119.5 (1,***P***< .001) Missing: 0**	42.8 (74)	57.2 (99)	**12.7 (1,***P***< .001) Missing: 0**	40.0 (186)	60.0 (279)	**57.9 (1,***P***< .001) Missing: 0**	54.2 (130)	45.8 (110)	**54 (1,***P***< .001) Missing: 0**
		**Difficulty speaking**			**Difficulty speaking**			**Difficulty speaking**			**Difficulty speaking**			**Difficulty speaking**			**Difficulty speaking**		
	Healthy	6.4 (53)	93.6 (779)	**69 (2,***P***< .001) Missing: 38**	3.4 (18)	96.6 (519)	**96.4 (2,***P***< .001) Missing: 3**	17.0 (90)	83.0 (439)	5.6 (2, *P* = .061) Missing: 3	4.2 (13)	95.8 (293)	4.3 (2, *P* = .114) Missing: 10	6.5 (17)	93.5 (245)	**13.3 (2,***P***= .001) Missing: 27**	5.5 (19)	94.5 (325)	**35.8 (2,***P***< .001) Missing: 8**
	Gingivitis	7.9 (63)	92.1 (738)		11.5 (44)	88.5 (337)		11.9 (29)	88.1 (215)		7.8 (16)	92.2 (190)		7.8 (25)	92.2 (297)		8.3 (27)	91.7 (297)	
	Periodontal disease	19.3 (110)	80.7 (461)		33.3 (36)	66.7 (72)		19.7 (44)	80.3 (179)		8.5 (16)	91.5 (173)		13.5 (89)	86.5 (571)		23.8 (29)	76.2 (93)	
	Spitting, seeing blood	14.2 (130)	85.8 (786)	**23.4 (1,***P***< .001) Missing: 0**	25.7 (54)	74.3 (156)	**80.3 (1,***P***< .001) Missing: 0**	36.0 (91)	64.0 (162)	**95.8 (1,***P***< .001) Missing: 0**	9.8 (17)	90.2 (156)	**4.7 (1,***P***= .030) Missing: 0**	13.1 (61)	86.9 (404)	**5.2 (1. (***P***= .023) Missing: 0**	14.6 (35)	85.4 (205)	**9 (1,***P***= .003) Missing: 0**
		**Embarrassed to smile or laugh**			**Embarrassed to smile or laugh**			**Embarrassed to smile or laugh**			**Embarrassed to smile or laugh**			**Embarrassed to smile or laugh**			**Embarrassed to smile or laugh**		
	Healthy	12.3 (102)	87.7 (730)	**30.7 (2,***P***< .001) Missing: 38**	6.7 (36)	93.3 (501)	**118.7 (2,***P***< .001) Missing: 3**	17.4 (92)	82.6 (437)	**25.3 (2,***P***< .001) Missing: 3**	11.8 (36)	88.2 (270)	5.7 (2, *P* = .058) Missing: 10	4.6 (12)	95.4 (250)	**7.5 (2,***P***= .023) Missing: 27**	10.8 (37)	89.2 (307)	**46.9 (2,***P***< .001) Missing: 8**
	Gingivitis	13.0 (104)	87.0 (697)		17.8 (68)	82.2 (313)		16.0 (39)	84.0 (205)		14.6 (30)	85.4 (176)		7.1 (23)	92.9 (299)		18.2 (59)	81.8 (265)	
	Periodontal disease	22.2 (127)	77.8 (444)		47.2 (51)	52.8 (57)		32.3 (72)	67.7 (151)		19.6 (37)	80.4 (152)		9.8 (65)	90.2 (595)		38.5 (47)	61.5 (75)	
	Spitting, seeing blood	21.8 (200)	78.2 (716)	**53.5 (1,***P***< .001) Missing: 0**	33.8 (71)	66.2 (139)	**71.4 (1,***P***< .001) Missing: 0**	47.0 (119)	53.0 (134)	**147.7 (1,***P***< .001) Missing: 0**	24.9 (43)	75.1 (130)	**16 (1,***P***< .001) Missing: 0**	12.5 (58)	87.5 (407)	**19.7 (1,***P***< .001) Missing: 0**	28.3 (68)	71.7 (172)	**21.8 (2,***P***< .001) Missing: 0**
		**Problems sleeping**			**Problems sleeping**			**Problems sleeping**			**Problems sleeping**			**Problems sleeping**			**Problems sleeping**		
	Healthy	9.4 (78)	90.6 (754)	**50.1 (2,***P***< .001) Missing: 38**	3.5 (19)	96.5 (518)	**145 (2,***P***< .001) Missing: 3**	12.9 (68)	87.1 (461)	**10.3 (2,***P***= .006) Missing: 3**	7.2 (22)	92.8 (284)	**12.1 (2,***P***= .002) Missing: 10**	3.4 (9)	96.6 (253)	1.9 (2, *P* = .393) Missing: 27	8.4 (29)	91.6 (315)	**36.1 (2,***P***< .001) Missing: 8**
	Gingivitis	12.1 (97)	87.9 (704)		11.5 (44)	88.5 (337)		14.3 (35)	85.7 (209)		16.5 (34)	83.5 (172)		5.0 (16)	95.0 (306)		13.6 (44)	86.4 (280)	
	Periodontal disease	22.2 (127)	77.8 (444)		42.6 (46)	57.4 (62)		22.0 (49)	78.0 (174)		14.8 (28)	85.2 (161)		5.6 (37)	94.4 (623)		30.3 (37)	69.7 (85)	
	Spitting, seeing blood	21.3 (195)	78.7 (721)	**74.5 (1,***P***< .001) Missing: 0**	28.6 (60)	71.4 (150)	**90 (1,***P***< .001) Missing: 0**	33.6 (85)	66.4 (168)	**88.7 (1,***P***< .001) Missing: 0**	19.1 (33)	80.9 (140)	**11.6 (1,***P***< .001) Missing: 0**	8.4 (39)	91.6 (426)	**17.2 (1,***P***< .001) Missing: 0**	23.8 (57)	76.3 (183)	**26.8 (1,***P***< .001) Missing: 0**
		**Limited participation in social activities**			**Limited participation in social activities**			**Limited participation in social activities**			**Limited participation in social activities**			**Limited participation in social activities**			**Limited participation in social activities**		
	Healthy	7.8 (65)	92.2 (767)	**67.4 (2,***P***< .001) Missing = 38**	3.4 (18)	96.6 (519)	**109.6 (2,***P***< .001) Missing: 3**	20.0 (106)	80.0 (423)	**8 ( 2,***P***= .018) Missing: 3**	5.2 (16)	94.8 (290)	4.3 (2, *P* = .114) Missing: 10	5.7 (15)	94.3 (247)	**7.8 (2,***P***= .020) Missing: 27**	4.4 (15)	95.6 (329)	**30.3 (2,***P***< .001) Missing: 8**
	Gingivitis	11.2 (90)	88.8 (711)		8.7 (33)	91.3 (348)		13.1 (32)	86.9 (212)		9.2 (19)	90.8 (187)		5.3 (17)	94.7 (305)		6.8 (22)	93.2 (302)	
	Periodontal disease	22.4 (128)	77.6 (443)		34.3 (37)	65.7 (71)		22.9 (51)	77.1 (172)		4.8 (9)	95.2 (180)		9.7 (64)	90.3 (596)		19.7 (24)	80.3 (98)	
	Spitting, seeing blood	17.1 (157)	82.9 (759)	**24.3 (1,***P***< .001) Missing: 0**	23.3 (49)	76.7 (161)	**72 (1,***P***< .001) Missing: 0**	36.8 (93)	63.2 (160)	**70.3 (1,***P***< .001) Missing: 0**	8.1 (14)	91.9 (159)	1 (1, *P* = .319) Missing: 0	11.0 (51)	89.0 (414)	**11.6 (1,***P***< .001) Missing: 0**	12.1 (29)	87.9 (211)	**7.7 (1,***P***= .006) Missing: 0**
		**Difficulty carrying out major work/role**			**Difficulty carrying out major work/role**			**Difficulty carrying out major work/role**			**Difficulty carrying out major work/role**			**Difficulty carrying out major work/role**			**Difficulty carrying out major work/role**		
	Healthy	5.8 (43)	94.2 (694)	**50.4 (2,***P***< .001) Missing = 326**	2.7 (14)	97.3 (507)	**111.9 (2,***P***< .001) Missing: 41**	15.3 (78)	84.7 (432)	5.6 (2, *P* = .060) Missing: 85	3.1 (9)	96.9 (277)	**6 (2,***P***= .050) Missing: 55**	1.7 (4)	98.3 (229)	3.9 (2, *P* = .143) Missing: 211	4.9 (16)	95.1 (309)	**18.1 (2,***P***< .001) Missing: 52**
	Gingivitis	5.7 (40)	94.3 (662)		8.5 (31)	91.5 (332)		9.2 (20)	90.8 (198)		7.3 (14)	92.7 (179)		2.3 (6)	97.7 (258)		5.9 (18)	94.1 (287)	
	Periodontal disease	16.1 (77)	83.9 (400)		33.7 (35)	66.3 (69)		16.1 (30)	83.9 (156)		7.9 (14)	92.1 (163)		4.1 (23)	95.9 (540)		16.4 (19)	83.6 (97)	
	Spitting, seeing blood	13.4 (105)	86.6 (681)	**41.8 (1,***P***< .001) Missing: 293**	22.4 (44)	77.6 (152)	**68 (1,***P***< .001) Missing: 38**	33.8 (75)	66.2 (147)	**95.7 (1,***P***< .001) Missing: 83**	10.8 (18)	89.2 (148)	**11.7 (1,***P***< .001) Missing: 46**	3.6 (14)	96.4 (379)	.2 (1, *P* = .649) Missing: 190	10.3 (24)	89.7 (210)	**4 (1,***P***= .047) Missing: 44**

*Note*: Significant *P* values are shown in bold.

Periodontal status was significantly associated with discomfort, pain and difficulty eating in all countries, with higher proportions of those with periodontitis experiencing these effects than those with gingivitis, and higher proportions with gingivitis experiencing these effects than those with healthy teeth. Where other significant associations were present, this same pattern tended to be observed. Nonsignificant associations included difficulty speaking in India and Italy, feeling embarrassed to smile or laugh in Italy, problems sleeping in Japan, limited participation in social activities in Italy, and carrying out one’s major work or role in India and Japan.

## Discussion

### Summary of findings

This was the first study to report standardised international data to assess patterns associated with patient and dentist-reported measures related to gum health. Around a third of the patient sample self-reported spitting or seeing blood when brushing, while almost two thirds of the sample were found to have nonhealthy periodontal status (reported by the dentist). Spitting or seeing blood was significantly associated with periodontal status, with the prevalence increasing with disease severity.

Spitting or seeing blood was associated with age in China, Japan and Lebanon, as well as with education in five countries, with patterns across age groups and education levels varying by country. There was also a pattern of worsening periodontal status associated with age, lower levels of education in most countries (except India and Japan) and sex, with female participants experiencing better periodontal status in all countries expect Colombia and India.

In all six countries spitting or seeing blood and nonhealthy periodontal status were associated with poorer oral and general health, with gingivitis, periodontitis and spitting or seeing blood having a greater effect on self-rated oral health than self-rated general health.

Spitting or seeing blood was significantly associated with both agreement (China, India, Lebanon) and disagreement (Colombia, Japan) that oral health had a good impact on wellbeing. This statement was only associated with periodontitis in Colombia, India and Japan, with higher proportions of those with gingivitis and periodontal disease agreeing in India and disagreeing in Colombia and Japan. In all six countries higher proportions of participants spitting or seeing blood agreed life had been less satisfying in the past 12 months, with worsening patterns for this variable also seen for those with gingivitis and periodontitis.

Pain, discomfort and difficulties eating were associated with higher proportions of participants spitting or seeing blood and having gingivitis and periodontitis in all six countries. Spitting or seeing blood was also associated with difficulty speaking, being embarrassed to smile and laugh and problems sleeping in all six countries, and in the majority of countries with limited participation in social activities (excluding Italy) and difficulty with carrying out work (excluding Japan). Oral health-related impacts were also associated with worsened periodontal status in the majority of cases.

## Interpretation

This descriptive analysis has demonstrated the different patterns associated with gum health, both patient and dentist-reported, with socio-demographic variables, self-rated oral and general health, wellbeing and life satisfaction, and oral health-related impacts. Some of the results of this research are in line with previous studies. The worsening of periodontal status with age has been documented previously in the literature,^35^ as well as with socio-demographic variables such as education.^36^ Previous research has also demonstrated better oral health outcomes in females, a pattern also generally present in these findings.^37^ Additionally, previous studies have made links between self-rated and clinical periodontal health and worsened oral health^38^^,^^39^ and general health.^40^^,^^41^ It is also not surprising that both the patient and dentist-reported measures affected self-rated oral health to a greater extent than self-rated general health, given that these may be two of many factors affecting health more generally, while proportionally they are likely to be more important in the context of oral health. In line with previous research,^28^ the importance of self-rated oral health-related impacts was again emphasised in this research, pointing to the necessity of considering these in evaluating the needs of patients.

Interestingly, patterns associated with spitting or seeing blood when brushing were less consistent than patterns associated with dentist-reported periodontal status. Nevertheless, self-reported data are of vital importance, with previous research demonstrating that self-impression and self-awareness of oral health are highly associated with both periodontal stages and presence,^42^ and that self-reported outcomes are considered central in understanding what it means to live with a condition.^43^ It may be that patients interpret self-rated questions in different ways depending on their experiences and perspective.^43^ It was also interesting to note that responses to questions on the impact of oral health on wellbeing and life satisfaction resulted in differing patterns. This may reflect that these are two differing concepts^44^ which have different meanings to participants, which may particularly be the case in the different countries (and contexts) in which the data were collected.

Although some variables were significant in multiple countries, the patterns associated with these variables were different across countries (eg, the association between spitting or seeing blood and age in China, Japan and Lebanon). These differences point to country-specific patterns and contexts which were unable to be accounted for in this research, as it was felt to be inappropriate to comment on potential contextual factors (such as healthcare systems and other characteristics of participating countries). At this point, the analysis can only be exploratory, and future work is required to understand the national and regional contexts in more detail with the input of NDAs and other collaborators in these countries. Once context is accounted for, direct comparison of patterns between countries may also be possible. This may also help to assess demographic and socio-economic differences between the countries which may have implications for differences found in the study’s findings. Accounting for context may also help in understanding some of the more counterintuitive findings from the study, such as the associations in some countries between periodontal status and both education and impacts on general wellbeing.

## Strengths and limitations

The main strengths of the analysis were the use of standardised and comparable international data, and the inclusion and analysis of countries which have been underrepresented in the oral health literature to date. Two of these countries (Japan and China) have both national and OHO datasets, and individual countries are conducting their own analyses which can contextualise the findings.^45^, ^46^, ^47^ The questionnaires used to collect data as part of the OHO project were also based on socio-demographic and oral health-related variables which were found to be important based on existing literature and the ICHOM AOHSS.^16^

More generally, the OHO project is also beneficial in its potential to deliver near real time data from different settings and could potentially act as a lower cost alternative to national epidemiological surveys which can have much larger intervals between data collection. Recent data collection from two additional countries with limited national data (Kenya and Tanzania) will add to the scope of the project, and it is hoped that further workshops and publications using the data^28^ can also aid in advocacy and policy-related discussions.

The study also has limitations. While data were standardised, it is possible that some missing data on oral health-related impacts in Colombia may have affected that analysis. Additionally, due to the nature of data collection (in dental practices), conclusions can only be drawn about those who attend the dentist, and groups with certain characteristics may be overrepresented in the data, as these may not be nationally representative. Similarly, data were only collected in practices which are part of their respective NDA meaning that this data may not even be representative of all dental practices in a given country. As a result of collecting data from patients attending dental practices, there are likely to be underestimates and overestimates in the data collected compared to what might be seen for the overall population in a given country. Future work on the issue of the representativeness of the data would be beneficial. Additionally, while it would be interesting and extremely valuable for the next steps of the project to predict models of oral health outcomes for the countries involved, this paper was focused on description of the data and assessing the prevalence of gingival health. Future papers should use multivariate regression to account for confounders and allow for prediction to be included in the analysis.

Regarding clinical variables, no formal training or calibration of the clinical data was conducted which would have allowed for uniform interpretation of the clinical criteria. This did reflect a more pragmatic approach about the reporting of oral diseases in the different countries included in this study, which has value in itself. Additionally, the way the gum health data was categorised in this study may have led to a loss of sensitivity in the variables and a loss of information, although the outcome does match previously published guidelines.^29^ The number of natural teeth for each patient was also not included in this analysis, which should be considered when interpreting the finding from the study.

## Conclusions

This was the first study to use standardised international data on oral health to assess the associations between gum health, socio-demographics and a variety of indicators of health, wellbeing, life satisfaction and oral health-related impacts. The findings demonstrated the differing patterns of associations that were important for gum health (both patient and dentist-reported) in each country, including associations with age, sex and education (to varying degrees), as well as the worsening of self-rated measures with increased symptoms and worsened clinical diagnosis in most cases. The lack of adjustment for potential confounding variables should be considered when interpreting these findings. These data can act as a starting point for advocacy and identifying the needs of patients, as well as further research into this field. Further research into country-specific patterns, particularly at subnational levels, as well as the addition of context for these patterns and the results demonstrated in this paper may provide additional insight.

## Author contributions

Tom Broomhead contributed to data linkage, interpretation and drafted and critically revised the manuscript. Jennifer Kettle contributed to data analysis and interpretation, and drafted and critically revised the manuscript. Steve Mason contributed to conception, design, data acquisition and interpretation and critically revised the manuscript. Sarah Baker contributed to data linkage, data analysis and interpretation, and drafted and critically revised the manuscript.

## Funding

Funding for the overall Oral Health Observatory project was provided by Haleon to FDI Central Office, which then coordinated and managed the research. The detailed analysis of the data from the Oral Health Observatory reported in this manuscript was separately funded by Haleon to University of Sheffield.

## Conflict of interest

None disclosed.

## References

[1] Buset S.L., Walter C., Freidmann A., Weiger R., Borgnakke W.S., Zitzmann NU. (2016). Are periodontal diseases really silent? A systematic review of their effect on quality of life. J Clin Periodontol.

[2] Wong B.L., Yap A.U., Allen PF. (2021). Periodontal disease and quality of life: umbrella review of systematic reviews. J Periodontal Res.

[3] Ferreira M.C., Dias-Pereira A.C., Branco-de-Almeida L.S., Martins C.C., Paiva SM. (2017). Impact of periodontal disease on quality of life: a systematic review. J Periodontal Res.

[4] O’Dowd L.K., Durham J., McCracken G.I., Preshaw P.M. (2010). Patients’ experiences of the impact of periodontal disease. J Clin Periodontol.

[5] Yin J., Li Y., Feng M., Li L. (2022). Understanding the feelings and experiences of patients with periodontal disease: a qualitative meta-synthesis. Health Qual Life Out.

[6] Wagner T.P., Costa R.S.A., Rios F.S. (2016). Gingival recession and oral health-related quality of life: a population-based cross sectional study in Brazil. Community Dent Oral Epidemiol.

[7] Goergen J., Costa R.S.A., Rios F.S. (2023). Oral conditions associated with oral health related quality of life: a population-based cross-sectional study in Brazil. J Dent.

[8] Brennan D.S., Spencer A.J., Roberts-Thomson KF. (2007). Quality of life and disability weights associated with periodontal disease. J Dent Res.

[9] Fuller J., Donos N., Suvan J., Tsakos G., Nibali L. (2020). Association of oral health-related quality of life measures with aggressive and chronic periodontitis. J Periodontal Res.

[10] Musurlieva N., Stoykova M. (2015). Evaluation of the impact of chronic periodontitis on individual’s quality of life by a self-developed tool. Biotechnol Equip.

[11] Broomhead T., Gibson B.J., Parkinson C.R., Vettore M.V., Baker SR. (2022). Gum health and quality of life—subjective experiences from across the gum health-disease continuum in adults. BMC Oral Health.

[12] Broomhead T., Gibson B.J., Parkinson C., Robinson P.G., Vettore M.V., Baker SR. (2023). Development and psychometric validation of the gum health experience questionnaire. J Clin Periodontol.

[13] Barbosa T., Gaviao M.B.D., Mialhe FL. (2015). Gingivitis and oral health-related quality of life: a systematic literature review. Braz Dent Sci.

[14] Tomazoni F., Zanatta F.B., Tuchtenhagen S., da Rose G.N., Del Fabro J.P., Ardenghi TM. (2014). Association of gingivitis with child oral health-related quality of life. J Periodontol.

[15] FDI World Dental Federation (2015). https://www.fdiworlddental.org/oral-healthatlas.

[16] Riordain R.N., Glick M., Al Mashhadani S.S.A. (2020). Developing a standard set of patient-centred outcomes for adult oral health - an international, cross-disciplinary consensus. Int Dent J.

[17] Guarnizo-Herrano C.C., Watt R.G., Pikhart H., Sheiham A., Tsakos G. (2013). Socioeconomic inequalities in oral health in different European welfare state regimes. J Epidemiol Comm Health.

[18] Guarnizo-Herrano C.C., Watt R.G., Pikhart H., Sheiham A., Tsakos G. (2014). Inequalities in oral impacts and welfare regimes: analysis of 21 European countries. Comm Dent Oral Epidemiol.

[19] Guarnizo-Herrano C.C., Watt R.G., Stafford M., Sheiham A., Tsakos G. (2017). Do welfare regimes matter for oral health? A multilevel analysis of European countries. Health Place.

[20] Guarnizo-Herrano C.C., Watt R.G., Garzon-Orjuela N., Tsakos G. (2018). Explaining oral health inequalities in European welfare state regimes: the role of health behaviours. Comm Dent Oral Epidemiol.

[21] Listl S., Moeller J., Manski R. (2014). A multi-country comparison of reasons for dental non-attendance. Eur J Oral Sci.

[22] Baker S.R., Foster Page L., Thomson W.M. (2017). Structural determinants and children’s oral health: a cross-national study. J Dent Res.

[23] Cheng H.G., Philips MR. (2014). Secondary analysis of existing data: opportunities and implementation. Shanghai Arch Psychiatry.

[24] Kassebaum N.J., Bernabe E., Dahiya M., Bhandari B., Murray C.J.L., Marcenes W. (2014). Global burden of severe periodontitis in 1990- 2010: a systematic review and meta regression. J Dent Res.

[25] FDI World Dental Federation (2020). https://www.fdiworlddental.org/oral-health-observatory.

[26] Taylor S., Baker S.R., Broomhead T. (2023). Standardised practice-based oral health data collection: a pilot study in different countries. Int Dent J.

[27] NIHR Research Design Service London. Justify sample size for a feasibility study. Available from: https://www.rds-london.nihr.ac.uk/resources/justify-sample-size-for-a-feasibility-study/. Accessed 25 May 2022.

[28] Broomhead T., England R., Mason S. (2024). Using standardised international oral health-related datasets in 6 countries. Int Dent J.

[29] Sanz M., Herrera D., Kebschull M. (2020). Treatment of stage I–III periodontitis—the EFP S3 level clinical practice guideline. J Clin Periodontol.

[30] World Health Organization (2013).

[31] Office for National Statistics, Social Survey Division, Information Centre for Health and Social Care (2024).

[32] United Nations Educational, Scientific and Cultural Organization Institute for Statistics (2011). https://uis.unesco.org/sites/default/files/documents/international-standard-classification-of-education-isced2011-en.pdf.

[33] Borgonovi F., Pokropek A. (2017). Mind that gap: the mediating role of intelligence and individuals’ socio-economic status in explaining disparities in external political efficacy in 28 countries. Intell.

[34] IBM Corp (2021).

[35] Lopez R., Smith P.C., Gostemeyer G., Schwendicke F. (2017). Ageing, dental caries and periodontal diseases. J Clin Periodontol.

[36] Boillot A., El Halabi B., Batty G.D., Rangé H., Czernichow S., Bouchard P. (2011). Education as a predictor of chronic periodontitis: a systematic review with meta-analysis population-based studies. Plos One.

[37] Lipsky M.S., Su S., Crespo C.J., Hung M. (2021). Men and oral health: a review of sex and gender differences. Am J Mens Health.

[38] Atala-Acevedo C., McGrath R., Glenister K. (2023). Self-rated oral health as a valid measure of oral health status in adults living in rural Australia. Healthcare.

[39] Al-Bitar K.M., Garcia J.M., Han S., Guentsch A. (2024). Association between periodontal health status and quality of life: a cross-sectional study. Frontiers in Oral Health.

[40] Yu Y.H., Steffensen B., Chasman D.I., Buring JE. (2024). Self-reported oral health is associated with systemic health outcomes and all-cause mortality. J Am Dent Assoc.

[41] Nguyen V.T.N., Furuta M., Zaitsu T. (2021). Periodontal health predicts self-rated general health: a time-lagged cohort study. Community Dent Oral.

[42] Deng K., Pelekos G., Jin L., Tonetti M.S. (2021). Diagnostic accuracy of self-reported measures of periodontal disease: a clinical validation study using the 2017 case definitions. J Clin Periodontol.

[43] Sischo L., Broder HL. (2011). Oral health-related quality of life: what, why, how, and future implications. J Dent Res.

[44] Ruggeri K., Garcia Garzon E., Maguire A., Matz S., Huppert F.A. (2020). Well-being is more than happiness and life satisfaction: a multidimensional analysis of 21 countries. Health Qual Life Out.

[45] Yu Y.Z., Du S., Ding M., Cui Z.Y., Liu Y., Si Y. (2022). Correlation analysis between self-perceived oral health and self-perceived general health among adults in China. Chin J Dent Res.

[46] Tamura K., Ogawa H. (2021). Japanese version of the oral health observatory project using a mobile application. Jpn Dent Sci Rev.

[47] Daou D., Choufani A., Mashmouchi M., Doumit M. (2025). Patient- and clinician-reported oral health data in primary dental care practices in Lebanon. Cureus.

